# Fluvoxamine for Outpatient Management of COVID-19 to Prevent Hospitalization: A Systematic Review and Meta-analysis

**DOI:** 10.1001/jamanetworkopen.2022.6269

**Published:** 2022-04-06

**Authors:** Todd C. Lee, Simone Vigod, Émilie Bortolussi-Courval, Ryan Hanula, David R. Boulware, Eric J. Lenze, Angela M. Reiersen, Emily G. McDonald

**Affiliations:** 1 Division of Infectious Diseases, Department of Medicine, McGill University Health Centre, Montréal, Québec, Canada; 2 Clinical Practice Assessment Unit, Department of Medicine, McGill University Health Centre, Montréal, Québec, Canada; 3 Division of Experimental Medicine, Department of Medicine, McGill University, Montréal, Québec, Canada; 4 Department of Psychiatry, Temerty Faculty of Medicine, University of Toronto and Women’s College Hospital, Toronto, Ontario, Canada; 5 Division of Infectious Diseases and International Medicine, Department of Medicine, University of Minnesota, Minneapolis; 6 Department of Psychiatry, School of Medicine, Washington University in St Louis, St Louis, Missouri; 7 Division of General Internal Medicine, Department of Medicine, McGill University Health Centre, Montréal, Québec, Canada

## Abstract

**Importance:**

Widely available and affordable options for the outpatient management of COVID-19 are needed, particularly for therapies that prevent hospitalization.

**Objective:**

To perform a meta-analysis of the available randomized clinical trial evidence for fluvoxamine in the outpatient management of COVID-19.

**Data Sources:**

World Health Organization International Clinical Trials Registry Platform and ClinicalTrials.gov.

**Study Selection:**

Studies with completed outpatient trials with available results that compared fluvoxamine with placebo were included.

**Data Extraction and Synthesis:**

The PRISMA 2020 guidelines were followed and study details in terms of inclusion criteria, trial demographics, and the prespecified outcome of all-cause hospitalization were extracted. Risk of bias was assessed by the Cochrane Risk of Bias 2 tool and a bayesian random effects meta-analysis with different estimates of prior probability was conducted: a weakly neutral prior (50% chance of efficacy with 95% CI for risk ratio [RR] between 0.5 and 2.0) and a moderately optimistic prior (85% chance of efficacy). A frequentist random-effects meta-analysis was conducted as a senstivity analysis, and the results were contextualized by estimating the probability of any association (RR ≤ 1) and moderate association (RR ≤ 0.9) with reduced hospitalization.

**Main Outcomes and Measures:**

All-cause hospitalization.

**Results:**

This systematic review and meta-analysis of 3 randomized clinical trials and included 2196 participants. The RRs for hospitalization were 0.78 (95% CI, 0.58-1.08) for the bayesian weakly neutral prior, 0.73 (95% CI, 0.53-1.01) for the bayesian moderately optimistic prior, and 0.75 (95% CI, 0.58-0.97) for the frequentist analysis. Depending on the scenario, the probability of any association with reduced hospitalization ranged from 94.1% to 98.6%, and the probability of moderate association ranged from 81.6% to 91.8%.

**Conclusions and Relevance:**

In this systematic review and meta-analysis of data from 3 trials, under a variety of assumptions, fluvoxamine showed a high probability of being associated with reduced hospitalization in outpatients with COVID-19. Ongoing randomized trials are important to evaluate alternative doses, explore the effectiveness in vaccinated patients, and provide further refinement to these estimates. Meanwhile, fluvoxamine could be recommended as a management option, particularly in resource-limited settings or for individuals without access to SARS-CoV-2 monoclonal antibody therapy or direct antivirals.

## Key Points

**Question:**

Is early administration of fluvoxamine associated with hospitalization in symptomatic adult outpatients with confirmed COVID-19?

**Findings:**

In this systematic review and bayesian meta-analysis of 3 clinical trials, which accounted for varying prior probabilities coupled with a frequentist sensitivity analysis, there was a high probability (94.1%-98.6%) that fluvoxamine was associated with a reduced risk for hospitalization, with a frequentist risk ratio of 0.75 (95% CI, 0.58-0.97).

**Meaning:**

These findings suggest that fluvoxamine, a widely available and inexpensive treatment for outpatients with COVID-19, was associated with a reduction in hospitalizations.

## Teaser

This systematic review and meta-analysis examines randomized clinical trial evidence for fluvoxamine to prevent hospitalization of patients with COVID-19.

## Introduction

Finding effective outpatient therapies for COVID-19 has been a major research undertaking since the beginning of the pandemic. While therapies, such as direct antivirals and engineered monoclonal antibodies, represent the current state-of-the art, there are challenges with availability, access, administration, and affordability in most areas of the world. Drug repurposing, or the use of presently available and affordable medications for the management of COVID-19 is an area of substantial ongoing research interest. The first medication to gain international interest for repurposing was hydroxychloroquine; however, it was demonstrated to be ineffective in randomized controlled trials.^1^ A variety of other candidate molecules have been the subject of randomized controlled trials with varying success.^2^

One such medication is fluvoxamine, which is a selective serotonin reuptake inhibitor (SSRI) that is also a potent activator of the sigma-1 receptor^3^ which decreases inflammation via reducing endoplasmic reticulum stress.^4^ In a murine sepsis model, fluvoxamine administration reduced mortality predominantly through sigma-1 activation.^5^ On this preclinical basis, the phase 2 STOP COVID 1 trial was conducted.^6^ This 152 patient double-blind, randomized placebo-control trial found fluvoxamine effective at preventing progression to severe COVID-19 defined as hypoxemia with dyspnea or hospital admission. Shortly thereafter, a benefit in preventing hospitalization or death was also reported in an uncontrolled 113-person prospective cohort.^7^ Later, an association with survival in COVID-19 for users of fluvoxamine was identified in a multicenter retrospective cohort study.^8^

Two larger phase 3 trials have been subsequently completed: the STOP COVID 2 trial in the US and Canada^9^ and the TOGETHER trial in Brazil^10^ Both trials were presented to the US National Institutes of Health in August 2021. The STOP COVID 2 trial was stopped for futility in May 2021 after an interim analysis found that the low event rate seen in the trial was associated with a less than 10% conditional probability of demonstrating efficacy within an attainable sample size based on recruitment rate.^11^ The TOGETHER trial, which had a higher primary outcome event rate, was stopped after demonstrating clinical benefit.^10^ We conducted a systematic review and meta-analysis of outpatient fluvoxamine trials to contextualize the evidence regarding hospitalization to inform clinical decision making, policy, and guidelines.

## Methods

This systematic review and meta-analysis is reported according to Preferred Reporting Items for Systematic Reviews and Meta-analyses (PRISMA) 2020 reporting guideline.^12^ Study details in terms of inclusion criteria, trial demographics, and the prespecified outcome of all-cause hospitalization were extracted.

### Search Strategy, Study Selection, Data Extraction, and Assessment of Bias

On November 12, 2021, we searched the World Health Organization International Clinical Trials Registry Platform and ClinicalTrials.gov for all registered clinical trials of fluvoxamine for the treatment of patients with COVID-19. Two independent reviewers screened results for eligibility, which included completed studies of outpatients comparing fluvoxamine to placebo or standard of care. Studies with published or publicly presented data (with the authors’ permission) were selected for inclusion.

For included studies, we summarized study inclusion criteria and patient demographics. We selected all-cause hospitalization as the primary outcome of interest given the implications for health care resource use. Two reviewers extracted hospitalization outcome data in each treatment group and these numbers were subsequently verified with the study principal investigators by email. For the TOGETHER trial, the published primary outcome included hospitalization or emergency department observation lasting 6 hours or more. To arrive at a more homogeneous outcome, we contacted the TOGETHER trial authors and obtained outcome data on emergency department visits lasting more than 24 hours and used this as a more representative proxy for hospital admission than an ED visit alone. For the STOP COVID 2 trial, we obtained demographic and outcomes data directly from the principal investigators (EJL and AMR) based on their presentation to the National Institutes of Health.^11^ We only included intention-to-treat analyses even if per protocol analyses suggested larger effect sizes were possible. Two independent reviewers assessed each study for bias using the Cochrane risk-of-bias 2 tool for randomized trials.

### Statistical Analysis

We conducted 2 analyses with a bayesian random effects meta-analysis on the log RR scale using the bayesmeta^13^ and metafor^14^ packages in R version 4.1.1 (eMethods in the Supplement).^15^ We selected priors based on 2 estimates of how promising the pre-existing data were. Scenario 1 used a weakly informative neutral prior, given that most treatments in medicine have a RR (RR) between 0.5 and 2 and because the STOP COVID 2 trial results were neutral (with a population mean [μ] [SD] of 0 [0.355]) on the log RR scale, this corresponds to a 50% chance of efficacy with 95% probability that the RR would be between 0.5 and 2).^16^ Scenario 2 used a moderately optimistic prior given the positive results of the STOP COVID 1 trial and the prospective cohort (with a μ [SD] of −0.41 [0.4], which corresponds to a 85% chance the RR would be ≤1).^16^ In both scenarios, the prior for the heterogeneity parameter (τ) used a half-Cauchy distribution with scale 0.10, which is the mean heterogeneity for meta-analyses of trials using hospitalization outcomes.^17^ As a sensitivity analysis, we also conducted a frequentist restricted maximum likelihood (REML) random effects meta-analysis on the log RR scale using the metan^18^ module for STATA version 17 (StataCorp). Results were exponentiated to the RR scale for presentation.

Using the estimated RRs from the above analyses as the mean and their associated 95% CIs to calculate standard errors, we then simulated 100 000 trials in STATA version 17 (StataCorp) on the log scale. We generated a representative probability density function on the RR scale using kernel density estimation and graphed it with the relevant prior probabilities. We then estimated the probabilities of any association (RR < 1) and moderate association (RR ≤ 0.9) by integrating the area under the curves (eMethods in the Supplement).^19^ Given the low cost of fluvoxamine and decades of established safety, we decided in advance that a moderate association would correlate to an absolute risk reduction between 0.5% and 1% assuming a 5% to 10% baseline risk of hospitalization, as observed in the control groups of several outpatient clinical trials. For context, this corresponds to a number needed to treat of 100 patients to 200 patients. Two independent reviewers assessed the certainty of evidence for hospitalization using GRADE methods (Grading of Recommendations, Assessment, Development and Evaluations).^20^

## Results

**Figure 1.**
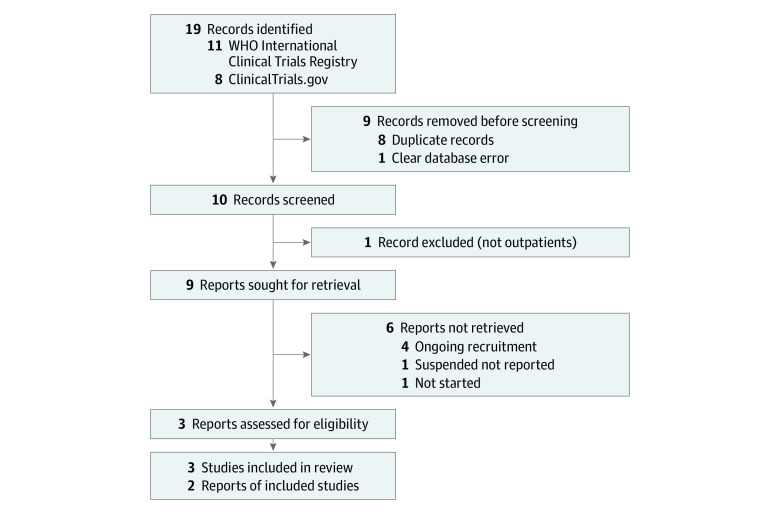
PRISMA Flow Diagram of Included Studies

The initial search yielded 19 candidate randomized controlled trials and 10 were retained following removal of duplicates (Figure 1; eTable 1 in the Supplement). Seven studies were then excluded for the following reasons: 4 studies because they were still recruiting participants,^21,22,23,24^ 1 study because it recruited only inpatients,^25^ 1 study because it was suspended without results,^26^ and 1 study was excluded because it was not yet recruiting.^27^

### Included Studies

The remaining 3 trials (STOP COVID 1 trial [N = 152],^6^ STOP COVID 2 trial [N = 547], and TOGETHER trial [N = 1497])^10^ included a total of 2196 analyzed patients (Table 1). It should be noted that because of its sample size, the TOGETHER trial is heavily weighted in the results of analyses. All 3 were placebo-controlled randomized controlled trials that recruited unvaccinated, symptomatic adults with microbiologically confirmed SARS-CoV-2 infection who were within 6 to 7 days of infection and not requiring oxygen. Whereas the STOP COVID 1 trial took all patients, the STOP COVID 2 and TOGETHER trials enriched their population for at least 1 at-risk feature for deterioration. Overall, the median age of participants was between 46 and 50, 55% to 72% of participants were female, 44% to 56% were obese. Most patients in the STOP COVID trials self-reported being White individuals; in the TOGETHER trial, 96% self-reported as being multiracial individuals. No trials included vaccinated patients and all trials predated both the Delta and Omicron variants. The risk of bias was considered low for all trials by both reviewers.

**Table 1.** Fluvoxamine COVID-19 Trial Details

Source	Dates	Original outcome	Inclusion criteria	Demographics	Fluvoxamine target dose^**a**^	Duration, d
Stop COVID 1^6^ United States	April 10, 2020-August 5, 2020	Clinical deterioration: Hospitalization or new hypoxemia within 15 d	Age ≥18 unvaccinated positive test with: ≤7 d symptoms	Median age 46; 72% female; 70% White individuals; 56% BMI ≥ 30; 20% hypertension; 11% diabetes	100 mg	15
				Median 4 d of symptoms		
Stop COVID 2^9^ United States and Canada	December 22, 2020-May 21, 2020	Clinical deterioration: Hospitalization or new hypoxemia within 15 d	Age ≥30 unvaccinated positive test with: ≤6 d symptoms	Median age 47; 62% female; 73% White individuals; 44% BMI ≥ 30; 21% hypertension; 9% diabetes	100 mg	15
			Criterion for high risk	Median 5 d of symptoms		
TOGETHER^10^ Brazil	January 20, 2021-August 5, 2021	ED visit ≥6 h or hospitalization within 28 d	Age ≥18 unvaccinated positive test with: ≤7 d symptoms	Median age 50; 55% female; 96% mixed race; 51% BMI ≥ 30; 13% hypertension; 16% diabetes	100 mg	10
			Criterion for high risk	Mean 3.8 d of symptoms^b^		

Abbreviations: BMI, body mass index; ED, emergency department.

^a^ Target dose was to be taken orally twice a day.

^b^ Median not provided; missing data on approximately 23% of participants.

### Meta-analysis

**Figure 2.**
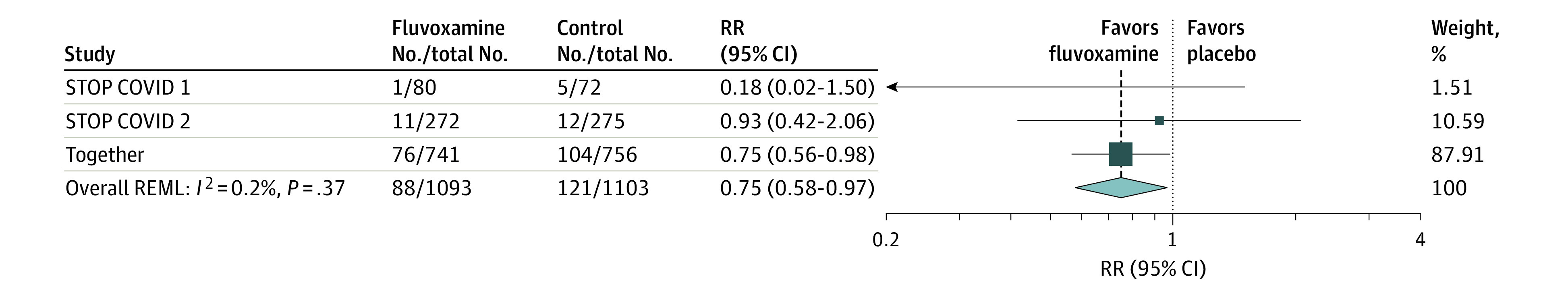
Frequentist Random Effects Meta-analysis

**Figure 3.**
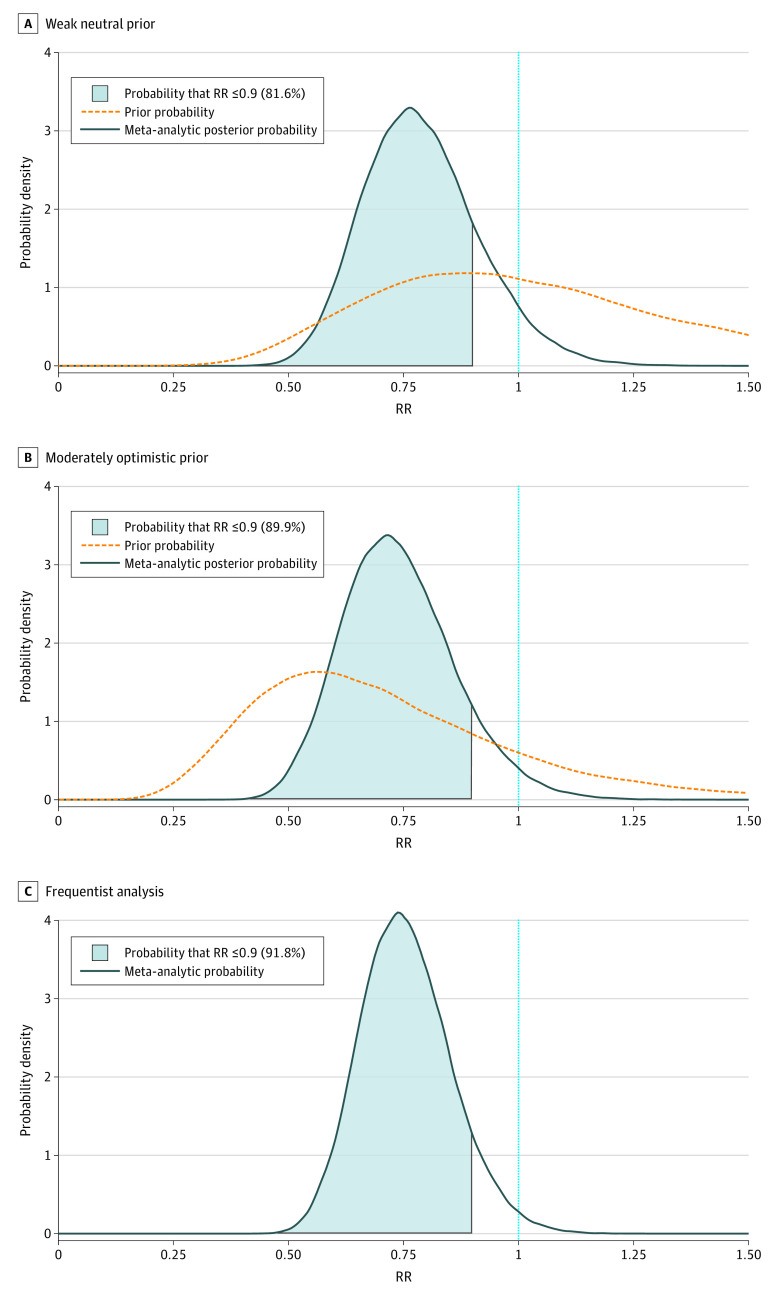
Probability Densities of the Primary and Sensitivity Analyses

In the bayesian analyses, the pooled RR in favor of fluvoxamine was 0.78 (95% CI, 0.58-1.08) for the weakly neutral prior and 0.73 (95% CI, 0.53-1.01) for the moderately optimistic prior (Table 2; eFigures 1 and 2 in the Supplement). In the frequentist meta-analysis, the pooled RR in favor of fluvoxamine was 0.75 (95% CI, 0.58-0.97; *I^2^*, 0.2%) (Figure 2). The overall probability of association with reduced hospitalization ranged from 94.1% to 98.6%, and of moderate association ranged from 81.6% to 91.8% (Table 2 and Figure 3). Statistical code needed to reproduce the analysis is included in the eMethods in the Supplement.

**Table 2.** Meta-analysis Results

Scenario	Pooled RR (95% CI)	Probability, %	
		RR < 1	RR ≤ 0.9
Weakly neutral	0.78 (0.58-1.08)	94.1	81.6
Moderately optimistic	0.73 (0.53-1.01)	97.2	89.9
Frequentist analysis	0.75 (0.58-0.97)	98.6	91.8

Abbreviation: RR, risk ratio.

### GRADE Certainty of Evidence

We felt the certainty of the evidence was moderate. Although all 3 trials were placebo-controlled randomized trials, there were some inconsistent findings given the STOP COVID 2 trial was terminated for futility. Although there was some risk of bias in the TOGETHER trial, we attempted to address this by limiting the primary outcome to emergency department visits that were 24 hours or longer, thus removing some concerns about the validity of shorter visits (6 to 24 hours in length). A moderate strength recommendation in favor of fluvoxamine could be considered in certain clinical scenarios as detailed in the discussion. According to the GRADE methods, such a recommendation is reasonable when shared decision-making is expected. Weaker recommendations usually apply when risks start to approach the benefits, or when significant resources are required for the intervention, neither of which is the case for fluvoxamine.

## Discussion

Based on currently available clinical trial data, we demonstrate high probability of an association between fluvoxamine and at least a moderate reduction in COVID-19 hospitalizations, under a variety of assumptions. By comparison, outpatient trials with hydroxychloroquine^1^ and ivermectin^28^ have not shown any efficacy and yet these agents continue to be prescribed. Further data from the ongoing identified studies such as the COVID-Out trial (NCT04510194) and the ACTIV-6 trial (NCT04885530) will help refine these estimates. Of note, both of these trials are using 50 mg twice daily of fluvoxamine, which, if unsuccessful, could indicate that 100 mg twice daily is the minimum effective dose. Recently, despite the results of the STOP COVID 1 trial and the TOGETHER trial, the Infectious Diseases Society of America recommended against the use of fluvoxamine, with the exception of use in clinical trial settings.^29^ Based on our analysis, and coupled with worldwide accessibility, decades of safety data, and a current price of approximately $1 per day,^30,31^ fluvoxamine may be a reasonable option for high-risk outpatients who do not have access to SARS-CoV-2 monoclonal antibodies, direct antivirals, or clinical trials. Even at a number to treat of 200 patients (absolute risk reduction, 0.5%), the corresponding cost to prevent admission would only be $2800. Clinicians who prescribe fluvoxamine for COVID-19 should familiarize themselves with relative contraindications and notable drug-drug interactions, including the need to limit caffeine (eTable 2 in the Supplement). The most common reported side effects from fluvoxamine in clinical trials are gastrointestinal (nausea, vomiting, diarrhea, and anorexia) and central nervous system complaints (headache, dizziness, somnolence, and nervousness).

Strengths of our analysis are the use of all available data and consistency of results by both a bayesian approach with multiple prior probability estimates and a classic random effects meta-analysis. For all analyses, we quantified the overall probability of any association and a moderate association of fluvoxamine with reduced hospitalization to help with decision-making. Further, we restricted the TOGETHER trial emergency department visits to those longer than 24 hours to address concerns^29^ about whether a 6-hour visit is an accurate proxy for health care use or marker of clinical deterioration. We used the intention-to-treat analysis, reflecting the reality of physician prescribing. In the TOGETHER trial, results were better in the per-protocol analysis.^10^

### Limitations

This review had limitations, including variability in health care practices, resource availability, and circulating variants between trials, leading to differences in the baseline event rates and the associated absolute risk reduction. While we have attempted to correct for subjectivity by limiting this analysis to hospitalizations, the decision to hospitalize may vary between geographic areas and even time points based on systemic burdens on the health care system. Nonetheless, all-cause hospitalization is the most common important outcome of outpatient COVID-19 trials because ICU admission or death would require prohibitively large studies. Additionally, all 3 trials excluded fully vaccinated individuals, whose rates of hospitalization are greatly reduced, and therefore any estimates of absolute effect size would likely be an overestimate in vaccinated patients. Another limitation is the inclusion of only 3 trials to date and the TOGETHER trial contributed between 66% and 88% of the analytic weight. Using a living systematic review approach will address this clinical question and incorporate emerging evidence.

## Conclusions

In this systematic review and meta-analysis of data from 3 trials, under a variety of assumptions, we found the probability that fluvoxamine was associated with reduced hospitalization ranged from 94.1% to 98.6% and the probability of moderate association ranged from 81.6% to 91.8%. Ongoing randomized controlled trials of fluvoxamine should continue, particularly those studying lower 50-mg doses (which may be better tolerated), evaluating efficacy in vaccinated individuals, or studying the related SSRI fluoxetine, which is on the World Health Organization’s list of essential medications. In the meantime, fluvoxamine is an immediately available, safe, and inexpensive management option with a high probability of moderate efficacy. It could be recommended as a treatment option for patients without contraindication, particularly in resource-limited settings or for individuals without access to monoclonal antibodies or direct antivirals.

## Notes

Supplement. **eMethods.** Statistical Code **eTable 1.** Details of Randomized Controlled Trials Identified in Search of Registry **eFigure 1.** Forest Plot of Bayesian Analysis With Weakly Neutral Prior **eFigure 2.** Forest Plot of Bayesian Analysis With Moderately Optimistic Prior **eTable 2.** Considerations for Relative Contraindications to Fluvoxamine
