# Supplementary material for: Fluvoxamine for Outpatient Management of COVID-19 to Prevent Hospitalization: A Systematic Review and Meta-analysis
Source: JAMA Netw Open. 2022 Apr 6;5(4):e226269. doi: 10.1001/jamanetworkopen.2022.6269 (PMC8987902; doi:10.1001/jamanetworkopen.2022.6269)
Supplement: Supplement. — eMethods. Statistical Code eTable 1. Details of Randomized Controlled Trials Identified in Search of Registry eFigure 1. Forest Plot of Bayesian Analysis With Weakly Neutral Prior eFigure 2. Forest Plot of Bayesian Analysis With Moderately Optimistic Prior eTable 2. Considerations for Relative Contraindications to Fluvoxamine [file jamanetwopen-e226269-s001.pdf]

Supplemental Online Content

Lee TC, Vigod S, Bortolussi-Courval É, et al. Fluvoxamine for outpatient management of COVID-19 to prevent hospitalization: a systematic review and meta-analysis. *JAMA Netw Open*. 2022;5(4):e226269. doi:10.1001/jamanetworkopen.2022.6269

eMethods. Statistical Code
eTable 1. Details of Randomized Controlled Trials Identified in Search of Registry
eFigure 1. Forest Plot of Bayesian Analysis With Weakly Neutral Prior
eFigure 2. Forest Plot of Bayesian Analysis With Moderately Optimistic Prior
eTable 2. Considerations for Relative Contraindications to Fluvoxamine

This supplemental material has been provided by the authors to give readers additional information about their work.

© 2022 Lee TC et al. *JAMA Network Open*.

eMethods. Statistical Code

R code

```
#add the libraries which are required (bayemeta will add its dependencies)

library(bayesmeta)

library(readxl)

#Import the data

Fluvox <- read_excel("Fluvox.xlsx")

#Set the seed to Jenny’s number

set.seed(8675039)

#Using escalc from the metafor package prepare the data for meta analysis

flu.es <- escalc(measure="RR", ai=fh, n1i=ft, ci=ph, n2i=pt, slab=Study, data=Fluvox)

#Weak neutral prior

ma02 <- bayesmeta(y = flu.es[,"yi"], sigma = sqrt(flu.es[,"vi"]), labels = flu.es[,"Study"],
mu.prior.mean = 0, mu.prior.sd = 0.355,tau.prior = function(t){dhalfcauchy(t,scale=0.1)})

#moderately optimistic prior

ma03 <- bayesmeta(y = flu.es[,"yi"], sigma = sqrt(flu.es[,"vi"]), labels = flu.es[,"Study"],
mu.prior.mean = -0.41, mu.prior.sd = 0.4, tau.prior = function(t){dhalfcauchy(t,scale=0.1)})

#Generate forest plots and obtain the point estimate and 95%CI from them

forestplot(ma02, exponentiate=TRUE)

forestplot(ma03, exponentiate=TRUE)

#Obtain the approximate weights

ma02$weights

ma03$weights
```

© 2022 Lee TC et al. *JAMA Network Open*.

STATA Code

/*STATA was used for two tasks. To conduct the frequentist meta-analysis and to create the probability density plots. This is provided for transparency, but no warranty or support is implied*/

/*To conduct the frequentist meta-analysis with metan you need to create the following variables study, fluvoxaminehospitalization, placebohospitalization, fluvoxaminenothospitalization, and placebonothospitalization and give them the appropriate values from the source data as presented in Figure 2. The command to regenerate the figure is below. */

```
metan fluvoxaminehospitalization fluvoxaminenothospitalization placebohospitalization
placebonothospitalization , favours(Fluvoxamine better # Placebo better) counts
label(namevar=study) model(reml) forestplot(range(0.25 4) xlabel(0.25 0.5 1 2 4))
group2(Fluvoxamine)
```

/*Create the probability density graphs*/

/*Generate 100000 simulated patients*/

```
clear
set obs 100000
set seed 8675309
gen var1=_n
```

/*Generate variables representing the RR and bounds of 95%CI from the optimistic (opt), weak neutral (called skep below) */

```
gen opt=0.73
gen lci_opt=0.53
gen uci_opt=1.01
gen skep=0.78
gen lci_skep=0.58
gen uci_skep=1.08
```

/*Put them on the log scale and obtain a standard error by looking at the 95% CI */

```
gen lnopt=log(opt)
gen seopt=(log(uci_opt)-log(lci_opt))/3.92
gen lnskep=log(skep)
gen seskep=(log(uci_skep)-log(lci_skep))/3.92
```

/*Simulate 100000 patients within the distributions informed by the point estimate of the log RR and 95% CI and then re-exponentiate to the RR scale*/

```
gen sim_opt=rnormal(lnopt,seopt)
gen sim_skep=rnormal(lnskep,seskep)
gen esim_opt=exp(sim_opt)
gen esim_skep=exp(sim_skep)
```

© 2022 Lee TC et al. *JAMA Network Open.*

```
/* Estimate the probability density function from the 100000.  Go get a coffee if you don’t have STATA MP*/

kdensity esim_skep, generate(x_skep y_skep) n(100000) nograph
kdensity esim_opt, generate(x_opt y_opt) n(100000) nograph

/* Create variables needed for the prior probability curves on the log RR scale and convert to the RR scale (skep=weak neutral) */

gen sim_skep_ref=rnormal(0, 0.355)
gen esim_skep_ref=exp(sim_skep_ref)
gen sim_opt_ref=rnormal(-0.41,0.4)
gen esim_opt_ref=exp(sim_opt_ref)

/* Integrate the areas under the kernel density functions where any means RR<1 and goal means RR less than or equal to 0.9 */

integ y_skep x_skep if x_skep<1
integ y_skep x_skep if x_skep<=0.9
integ y_opt x_opt if x_opt<1
integ y_opt x_opt if x_opt<=0.9


/* Make the plots! */

twoway (kdensity esim_skep, range (0.0 1.5))  (area y_skep x_skep if x_skep<=0.9) (kdensity esim_skep_ref, range(0.0 1.5)), xline(1,lpattern(dash) lcolor(cranberry)) scheme(scientific) xlabel(0(0.25)1.5) legend(order(2 3 1) label(3 "Prior probability") label(1 "Meta-Analytic Posterior Probability") label(2 "Probability that RR <=0.9 [81.6%]") position(3)) xti(Relative Risk) yti(Probability Density) title("Weak Neutral Prior") xsize(8) ysize(3.5)

twoway (kdensity esim_opt, range (0.0 1.5))  (area y_opt x_opt if x_opt<=0.9) (kdensity esim_opt_ref, range(0.0 1.5)), xline(1,lpattern(dash) lcolor(cranberry)) scheme(scientific) xlabel(0(0.25)1.5) legend(order(2 3 1) label(3 "Prior probability") label(1 "Meta-Analytic Posterior Probability") label(2 "Probability that RR <=0.9 [89.9%]") position(3)) xti(Relative Risk) yti(Probability Density) title("Moderately Optimistic Prior") xsize(8) ysize(3.5)


/* Do the same for the frequentist result */

clear
set obs 100000
set seed 8675309
gen var1=_n
gen freq=0.75
```

© 2022 Lee TC et al. *JAMA Network Open*.

```
gen lci_freq=0.58
gen uci_freq=0.97
gen lnfreq=log(freq)
gen sefreq=(log(uci_freq)-log(lci_freq))/3.92
gen sim_freq=rnormal(lnfreq , sefreq )
gen esim_freq=exp(sim_freq)
kdensity esim_freq, generate(x_freq y_freq) n(100000) nograph k(gaussian)
integ y_freq x_freq if x_freq<1
integ y_freq x_freq if x_freq<=0.9

// Plot

twoway (kdensity esim_freq, range (0 1.5))  (area y_freq x_freq if x_freq<=0.9),
xline(1,lpattern(dash) lcolor(cranberry)) scheme(scientific) xlabel(0(0.25)1.5) legend(order(2 1)
label(1 "Meta-Analytic Probability") label(2 "Probability that RR <=0.9 [91.8%]") position(3))
xti(Relative Risk) yti(Probability Density) title("Frequentist Analysis") xsize(8) ysize(3.5)
```

© 2022 Lee TC et al. *JAMA Network Open*.

eTable. Details of Randomized Controlled Trials Identified in Search of Registry

Title	Registration ID(s)	Countries	Outpatient	Maximum Daily Dose and Duration	Comparator	Status	Results Available	Included in meta-analysis
Effect of fluvoxamine on cytokine in COVID-19 patients	IRCT20131115015405N4	Iran	No	300mg x Not Specified	Standard of Care	Completed	NA	No
A Double-blind, Placebo-controlled Clinical Trial of Fluvoxamine for Symptomatic Individuals With COVID-19 Infection (STOP COVID)	NCT04342663	USA	Yes	300mg x15 days	Placebo	Completed	Yes	Yes
COVID-OUT: Early Outpatient Treatment for SARS-CoV-2 Infection (COVID-19)	NCT04510194	USA	Yes	100mg x10 days	Placebo	Recruiting	No	No
Fluvoxamine for Early Treatment of Covid-19 (Stop Covid 2)	NCT04668950	USA and Canada	Yes	200mg x15 days	Placebo	Completed	Yes	Yes
Fluvoxamine for Adults With Mild to Moderate COVID-19	NCT04711863	Republic of Korea	Yes	200mg x10days	Placebo	Suspended	No	No
Fluvoxamine Administration in Moderate SARS-CoV-2 (COVID-19) Infected Patients	NCT04718480 and EUCTR2020-002299-11-HU	Hungary	Yes	200mg x74 days	Placebo	Recruiting	No	No
Repurposed Approved and Under Development Therapies for Patients With Early-Onset COVID-19 and Mild Symptoms (TOGETHER)	NCT04727424	Brazil	Yes	200mg x10 days	Placebo	Completed	Yes	Yes
ACTIV-6: COVID-19 Study of Repurposed Medications	NCT04885530	USA	Yes	100mg x10 days	Placebo	Recruiting	No	No
Randomized-controlled Trial of the Effectiveness of COVID-19 Early Treatment in Community	NCT05087381	Thailand	Yes	150mg x14 days	Standard of Care	Recruiting	No	No
Effect of Combined Fluvoxamine with Favipiravir versus Favipiravir Monotherapy in Prevention of Clinical Deterioration among mild to moderate COVID-19 patients Monitoring by Telemedicine in Virtual Clinic: Open-label Randomized Controlled Trial	TCTR20210615002	Thailand	Yes	200mg x 10 days	Favipiravir	Not Started	NA	No

© 2022 Lee TC et al. *JAMA Network Open*.

eFigure 1. Forest Plot of Bayesian Analysis With Weakly Neutral Prior

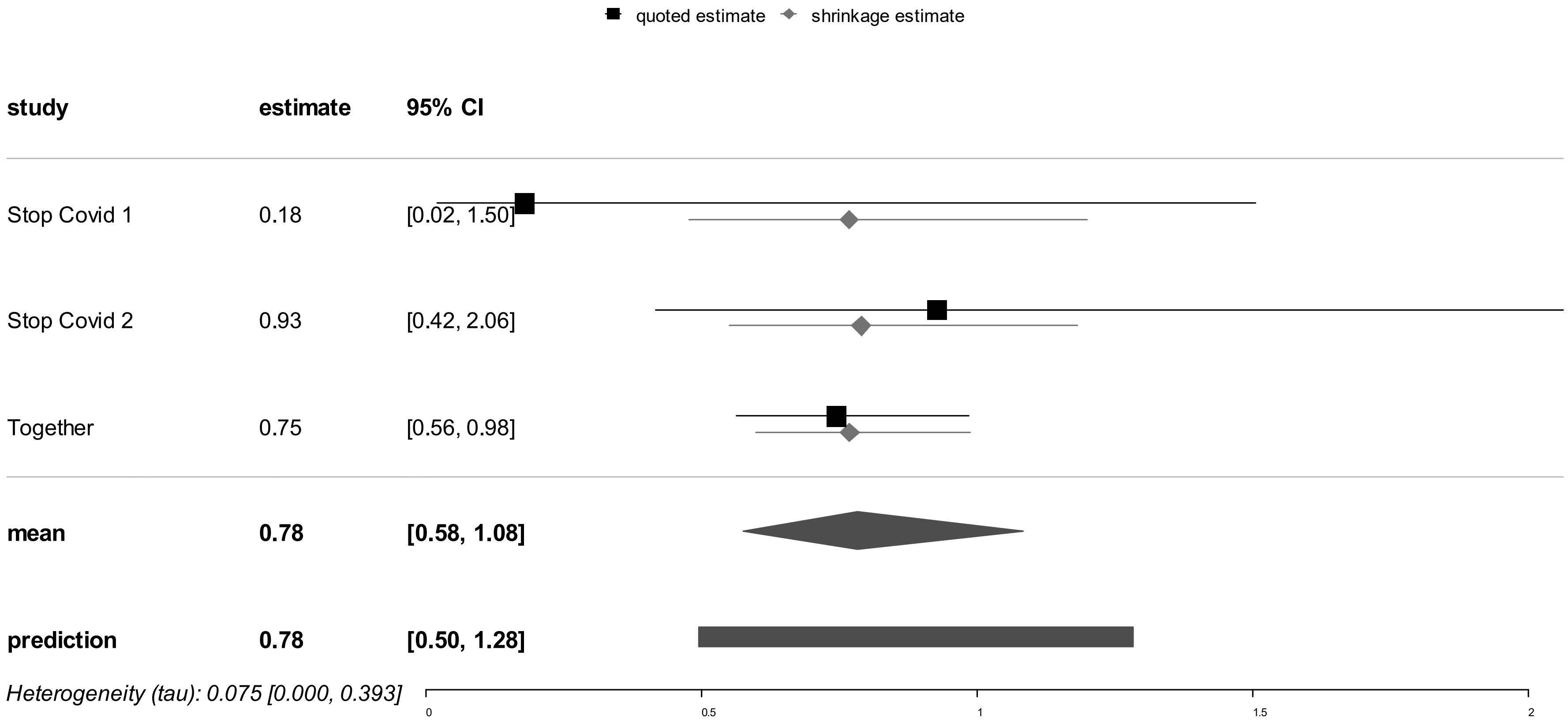

The approximate meta-analytic weights given for the mean effect are: 2.0% for Stop Covid 1, 11.9% for Stop Covid 2, 19.7% given to prior, and 66.3% given to Together

© 2022 Lee TC et al. *JAMA Network Open*.

eFigure 2. Forest Plot of Bayesian Analysis With Moderately Optimistic Prior

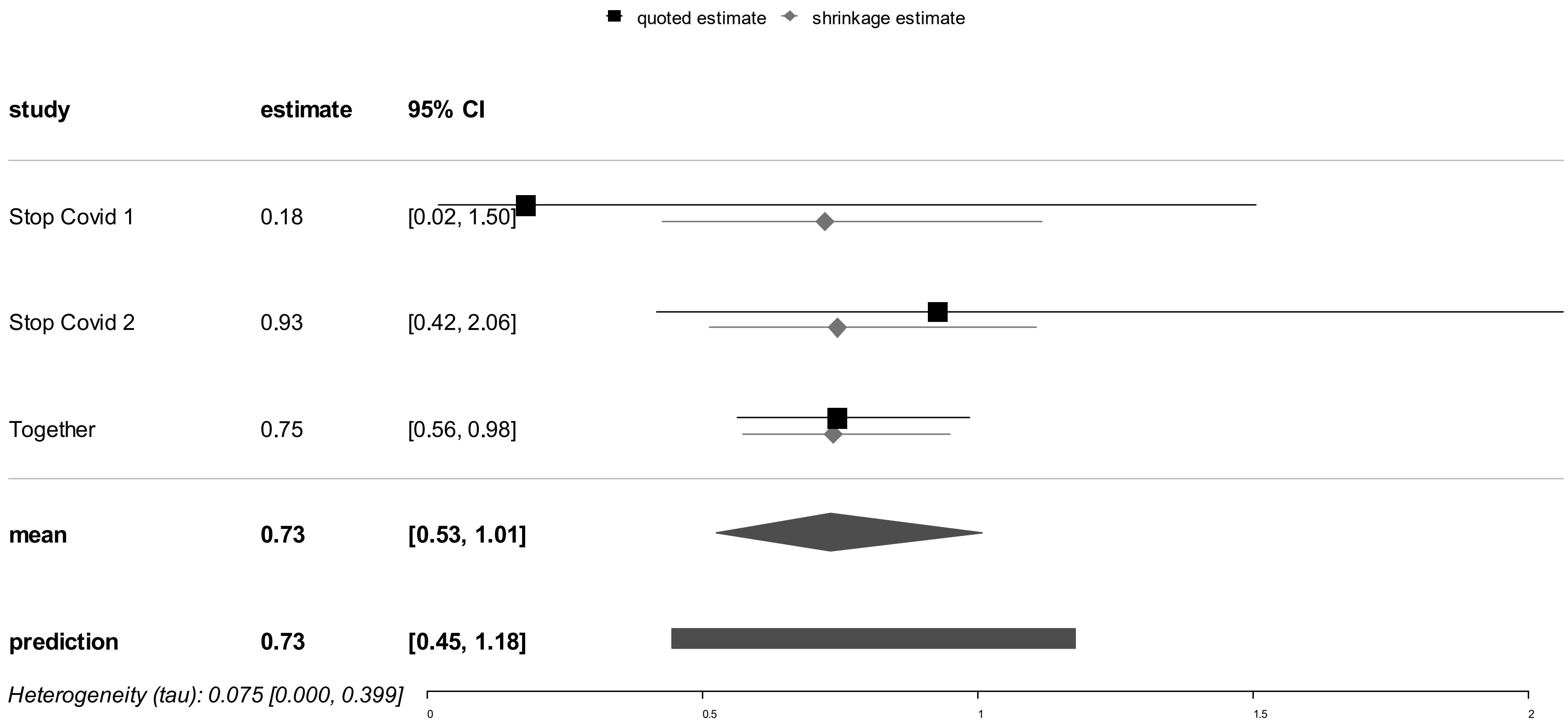

The approximate meta-analytic weights given for the mean effect are: 2.1% for Stop Covid 1, 12.5% for Stop Covid 2, 16.6% given to prior, and 68.8% given to Together

© 2022 Lee TC et al. *JAMA Network Open*.

eTable 2. Considerations for Relative Contraindications to Fluvoxamine

Patient Factors	Reasoning (if not obvious)
Allergy to fluvoxamine	
Moderate to severe depression within 6 weeks of enrollment	If the patient would need to be switched to fluvoxamine from another agent due to drug-interactions, this would ideally be done with explicit supervision
Previous or current diagnosis of manic depression / bipolar disorder	If the patient would need to be switched to fluvoxamine from another agent or if there would be concern that adding fluvoxamine might trigger a manic episode
Hepatic impairment defined as known Cirrhosis of any severity	Fluvoxamine metabolism is altered in patients with cirrhosis
Hospitalization for gastrointestinal or other non-traumatic bleeding within the last year	Fluvoxamine can impact platelet aggregation and these patients were excluded from the trial. This decision could be individualized.
Concurrent Medications	
Caffeine	Fluvoxamine leads to substantial increases in caffeine levels. In the trial, we encouraged no caffeine for participants. At the very least they were told avoid more than 1 small cup of coffee's worth of caffeine (and to stop caffeine if they felt it was "too energizing").
Patients taking warfarin	Increased bleeding risk due to increased AUC of warfarin
Patients taking clopidogrel	Increased risk of ischemic event due to metabolism
Patients taking 2 or more of the following: aspirin, NSAIDS, ticlopidine, prasugrel, ticagrelor, direct oral anticoagulants	Assuming NSAIDs cannot be held. Fluvoxamine can impact platelet aggregation and these patients were excluded from the trial. This decision could be individualized.
Donepezil	This is a Sigma-1-receptor (S1R) agonist and we excluded patients from the trial given that fluvoxamine was being used for its S1R activity
Other antidepressant medications	For any patient already on a tricyclic antidepressant, SSRI, or SNRI, we evaluated whether it could be held or reduced under medical supervision during the time they were prescribed fluvoxamine. If the patient was taking a low dose of another medication (e.g., citalopram 10mg) and there was low risk of serotonin syndrome, concurrent use was allowed.
Use within 14 days of an MAO inhibitor [e.g., Isocarboxazid (Marplan), Phenelzine (Nardil), Selegiline (Emsam), Tranylcypromine (Parnate)]	Important drug interactions risking serotonin syndrome

© 2022 Lee TC et al. *JAMA Network Open*.

Patient Factors	Reasoning (if not obvious)
Patients taking astemizole, cisapride, mesoridazine, ramelteon, or terfenadine	Contraindicated due to hepatic CYP3A4 interaction
Patients taking phenytoin or valproic acid	Potential interaction leading to seizure
Patients who are taking mirtazapine, melatonin, tramadol, or triptan medications	If these drugs could not be held, there was a risk of drug interaction increasing levels of these medicines
Participants taking alosetron, clozapine, flutamide, mexiletine, olanzapine, rasagiline, ropinirole, tacrine, theophylline, tizanidine, triamterene	Drugs are primarily metabolized by CYP1A2, which is inhibited by fluvoxamine.
Diazepam or alprazolam users	Due to interactions, we recommend reducing the dose by 25% unless the patient has a known seizure disorder (in which case they were excluded).

© 2022 Lee TC et al. *JAMA Network Open*.
